# Editorial: Insects as a model in behavioral ecology

**DOI:** 10.3389/finsc.2023.1298274

**Published:** 2023-10-04

**Authors:** Craig D. Perl, Emily Baird

**Affiliations:** ^1^ School of Life Sciences, Arizona State University, Tempe, AZ, United States; ^2^ Department of Zoology, Stockholm University, Stockholm, Sweden

**Keywords:** insects, insects and climate warming, behavioral ecology, animal monitoring, microCT (μCT) scanning technology, pollinator, climate change

Behavioral ecology is the study of the fitness consequences of behavior. The choices that animals make are, hopefully adaptive and lead to positive fitness gains. Insects are already widely employed as models for behavioral ecology, due to their easily manipulable size and the impressively wide range of behaviors they exhibit. While a lot is known about some groups, other species are severely under-investigated. Even for species that are well-known, many of the observations on their behavior are made, for good reason, under unnatural conditions in the lab. While this paradigm is extremely powerful at exploring how few variables might impact particular behaviors, it is limited at garnering a holistic view of the myriad factors that modulate behaviors in the field. Very little is known comparatively about how many behaviors function in the field, a knowledge gap that must be urgently met if the ways in which behavior and the environment interact are to be fully understood.

What happens when the behavior no longer matches the environment? In our rapidly changing world, understanding animal behavior in a natural context is of paramount importance for understanding ways in which we can mitigate the harmful impacts of environmental stressors such as light pollution, climate change and pesticide usage. The evolutionary pressures that organisms are adapted to are likely to change very rapidly, and therefore understanding the behavioral responses to a suddenly stressful environment is paramount for mitigation.

Insects are at the forefront of these rapidly changing environmental stressors with extremely alarming reports regarding pollinator (1, 2) and broad insect declines (3–5) being reported worldwide. Many of these are insects are vital for the function of many ecosystems and biomes (2, 6), therefore a deep understanding of their behavioral ecology is a crucial part of determining how to preserve these ecosystems. For eusocial pollinators in particular, many environmental stressors can manifest as reduced numbers of foragers within a given colony – a condition that may prove fatal and likely detrimental. Gérard et al. demonstrate, under natural foraging conditions, that bumblebee workers have the behavioral flexibility to compensate for rapidly reduced colony sizes. This sets the ground work for further longer-term studies that ascertain the impact on colonies across an entire season and on the longevity of workers.

There are some aspects of behavioral ecology that rapidly evolving technology will help to unravel. The true adaptive value of behavior can only be shown in conjunction with other biological properties; such as physiology, morphology, neuroscience or anatomy. Jonsson outlines recent advances in µCT and how in conjunction with artificial intelligence, especially deep-learning, high contrast anatomical imaging has revolutionized our capacity to gather enormous datasets on microscopic and submicroscopic anatomical structures. The potential for analyzing insect sensory structures and nervous systems makes µCT and AI-driven tools invaluable for integrating our understanding of behavioral ecology and the neuroanatomy that drives those behaviors.

The ability for new technology and techniques to aid in our understanding of behavioral ecology is further exemplified by the study of Wallace et al., that outlines a new method for long-term insect monitoring: “Camfi”. Camfi was built to optimize long-term population monitoring and generate high-throughput behavioral observation of low-flying insects. The use of wildlife cameras provides enormous economic benefits compared with current methods such as lidar. Wallace et al. proceed to exhibit the utility of their new system in a second paper examining the evening flights of the endangered Bogong moth. Using Camfi, Wallace et al. discover that the seemingly random evening flights are not random at all, but are oriented relative to a nearby mountain. They also present the first recorded behavioral response of Bogong moths to an increasingly common environmental stressor – bushfires. Moths fled their aestivation site the day after the fire, further demonstrating the insights provided by cost effective, long-term behavioral monitoring.

The goal of this Research Topic was to highlight the latest advances in insect behavioral ecology including both novel findings and novel methods. We hope that this Research Topic will provide inspiration for future developments in the field.

## Author contributions

CP: Writing – original draft, Writing – review & editing. EB: Writing – review & editing.

## References

[1] Potts SG Biesmeijer JC Kremen C Neumann P Schweiger O Kunin WE. Global pollinator declines: trends, impacts and drivers. Trends Ecol Evol (2010) 25(6):345–53. doi: 10.1016/j.tree.2010.01.007 20188434

[2] Rhodes CJ. Pollinator decline–an ecological calamity in the making? Sci Prog (2018) 101(2):121–60. doi: 10.3184/003685018X15202512854527 PMC10365189 29669627

[3] van der Sluijs JP. Insect decline, an emerging global environmental risk. Curr Opin Environ Sustain (2020) 46:39–42. doi: 10.1016/j.cosust.2020.08.012

[4] Hallmann CA Sorg M Jongejans E Siepel H Hofl and N Schwan H. More than 75 percent decline over 27 years in total flying insect biomass in protected areas. PloS One (2017) 12:e0185809. doi: 10.1371/journal.pone.0185809 29045418 PMC5646769

[5] Wagner DL Grames EM Forister ML Berenbaum MR Stopak D. Insect decline in the Anthropocene: Death by a thousand cuts. Proc Natl Acad Sci USA (2021) 118(2):e2023989118. doi: 10.1073/pnas.2023989118 33431573 PMC7812858

[6] Goulson D. The insect apocalypse, and why it matters. Curr Biol (2019) 29(19):R967–71. doi: 10.1016/j.cub.2019.06.069 31593678

